# Complex I activity in hypoxia: implications for oncometabolism

**DOI:** 10.1042/BST20230189

**Published:** 2024-03-25

**Authors:** Christos Chinopoulos

**Affiliations:** Department of Biochemistry, Semmelweis University, Budapest 1094, Hungary

**Keywords:** cancer, hypoxia, mitochondria, mtSLP, OXPHOS

## Abstract

Certain cancer cells within solid tumors experience hypoxia, rendering them incapable of oxidative phosphorylation (OXPHOS). Despite this oxygen deficiency, these cells exhibit biochemical pathway activity that relies on NAD^+^. This mini-review scrutinizes the persistent, residual Complex I activity that oxidizes NADH in the absence of oxygen as the electron acceptor. The resulting NAD^+^ assumes a pivotal role in fueling the α-ketoglutarate dehydrogenase complex, a critical component in the oxidative decarboxylation branch of glutaminolysis — a hallmark oncometabolic pathway. The proposition is that through glutamine catabolism, high-energy phosphate intermediates are produced via substrate-level phosphorylation in the mitochondrial matrix substantiated by succinyl-CoA ligase, partially compensating for an OXPHOS deficiency. These insights provide a rationale for exploring Complex I inhibitors in cancer treatment, even when OXPHOS functionality is already compromised.

## Background

It was only 13 years ago when Douglas Hanahan and Robert A Weinberg included in their seminal work ‘deregulating cellular energetics’ as a hallmark of cancer [1], 88 years after Warburg and Minami's landmark paper on aerobic glycolysis in tumors [2]. It is this author's opinion that addressing oncometabolism as ‘deregulated’ does no service, mindful of the extent and ingenuity of pathway rewiring. Instead, it should be branded as ‘evolution,’ albeit toward a sinister outcome for the host.

Metabolic reconfiguration contributing to cancer onset, growth and metastasis is a rapidly advancing field and will not be exhaustively covered here. The focus will be exclusively on the operation of Complex I of the respiratory chain in hypoxia. Other potential sources of mitochondrial NAD^+^ during acute hypoxia — including the recent discovery of SLC25A51 and its paralog, SLC25A52, both capable of mediating mitochondrial uptake of NAD^+^ [3] — are reviewed elsewhere [4]. The origin of Complex I substrate — ubiquinone (UQ) — will be examined. The fate of a Complex I product, NAD^+^, will be addressed as a necessary cofactor for the α-ketoglutarate dehydrogenase complex activity, yielding succinyl-CoA. It is this high-energy thioester that is used by succinyl-CoA ligase to harness high-energy phosphate intermediates through a process called mitochondrial substrate-level phosphorylation (mtSLP). The position of mtSLP within glutaminolysis will be highlighted. Eventually, the role of high-energy phosphate intermediates produced by mtSLP in hypoxia, not only supporting the reverse operation of F_0_–F_1_ ATP synthase but also maintaining the adenine nucleotide translocase (ANT) in the ‘forward’ mode supplying the cytosol with ATP, will be discussed.

## Rationale for Complex I targeting in cancer

Intratumoral hypoxia caused by hypoperfusion [5] has led to the proposal that OXPHOS in cancer cells is deficient [6–9]. This notion has not been universally accepted, with proponents arguing that some cancer cells exhibit normal OXPHOS [10,11]. However, the deeper dispute is whether cancer cell survival actually depends on OXPHOS. Our recent contribution to this was to show that depending on the cancer cell culture type and targeted inhibition of respiratory components, OXPHOS can be demonstrated but could be dispensable for cell survival [12]. Therefore, we proposed that the association between OXPHOS and tumor viability likely relies on the particular cell type and the precise means of respiratory inhibition. The latter part of this statement echoes a common misconception in the field of oncometabolism but also bioenergetics in general: although OXPHOS is a process dependent on the concerted action of several individual molecular entities, each entity may operate as stand-alone and participate in non-OXPHOS pathways. Thus, targeted inhibition of the electron transport chain will abolish OXPHOS (for notable exceptions see [13] and references therein), but the remaining complexes may still engage in bioenergetic activities. Essentially, ‘bioenergetics’ and ‘OXPHOS’ must not be used interchangeably.

Thus, mindful that OXPHOS maybe dispensable for cancer cell viability while its components could engage in other bioenergetic pathways, it is easy to envisage why targeting Complex I still makes sense as an anti-cancer target [14–16] irrespective of OXPHOS functionality. Indeed, inhibition of Complex I slowed tumor growth under several experimental settings [17–23]. Importantly, the anti-cancer effects of Complex I inhibitors were abolished when the inhibitor-resistant *Saccharomyces cerevisiae* NADH dehydrogenase NDI1 was overexpressed, proving that Complex I assists cancer by providing NAD^+^ [16]. Thus, it is the catalytic function of Complex I and specifically its ability to oxidize NADH that is responsible for promoting cancer, and not a potential non-enzymatic interaction with an entity not even the provision of reduced UQ. However, at least one study demonstrated that a decrease in Complex I activity led to enhanced cell adhesion, migration, invasion and spheroid formation of tumor cells, albeit, the decrease in activity was not >40% [24]; along these lines, pharmacological and/or genetic manipulations of Complex I under some conditions were shown to favor pro-cancer events [25–29]. But perhaps the greatest deterrent in using Complex I inhibitors against cancer is drug toxicity: phase I trials with IACS-010759, a highly potent and selective small-molecule Complex I inhibitor with favorable attributes for clinical evaluation [14] were discontinued due to no establishment of recommended phase 2 dose and modest anti-tumor effects [30]. Despite that this toxicity is not surprising mindful that each and every normal cell harboring mitochondria is dependent on Complex I activity for OXPHOS, the notion that provision of NAD^+^ is critical for tumor growth begs the question: which entity/pathway is the NAD^+^ beneficiary in hypoxia?

## Complex I activity in hypoxia

**Figure 1.**
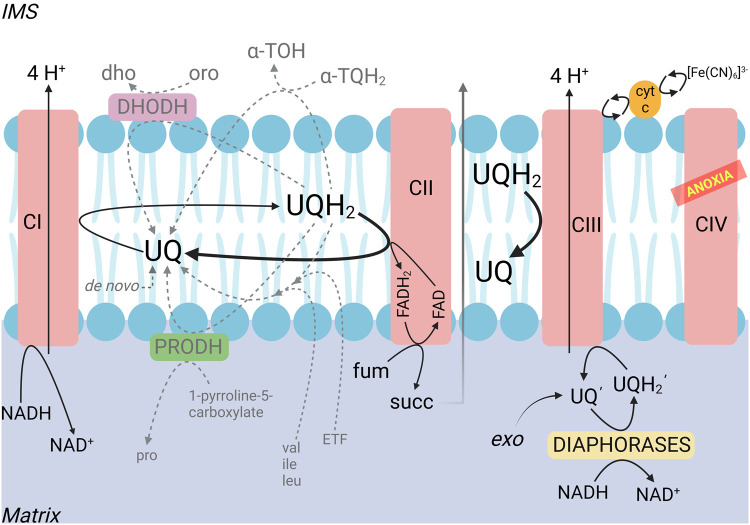
Sources of oxidized ubiquinone (UQ) within mitochondria. Solid lines indicate the main pathways. Gray dashed lines indicate minor, theoretical or impeded pathways as described in the text. [Fe(CN)_6_]^3−^, ferricyanide; α-TOH, alpha-tocopherol (biologically the most active form of vitamin E); α-TQH_2_, alpha-tocopheryl hydroquinone; CI, Complex I; CII, Complex II; CIII, Complex III; CIV, Complex IV; cyt c, cytochrome c; dho, dihydroorotate; DHODH, dihydroorotate dehydrogenase; ETF, electron transfer flavoprotein system; *exo*, exogenously added ubiquinone; fum, fumarate; ile, isoleucine; IMS, intermembrane space; leu, leucine; oro, orotate; pro, proline; PRODH, proline dehydrogenases; succ, succinate UQ, ubiquinone (oxidized) UQ′, ubiquinone’ (oxidized, amphiphilic, given exogenously); UQH_2_, ubiquinol (reduced); UQH_2_′, ubiquinol’ (reduced form of exogenously added amphiphilic ubiquinone’); val, valine. Created with BioRender.

To address Complex I operation during hypoxia, one needs to dwell on the availability of its substrates. Complex I catalyzes the oxidation of matrix NADH by UQ producing NAD^+^ and ubiquinol (UQH_2_), which is coupled to the translocation of four H^+^ across the mitochondrial inner membrane and the transfer of electrons downstream to FeS clusters [31]. NADH is generated in the mitochondrial matrix by hundreds of reactions (http://bit.ly/mNADH). Admittedly, many of those reactions are cell-specific (such as those related to the metabolism of steroids) but mindful of the reductive stress in hypoxia, NADH is not constraining. Oxidized UQ, on the other hand, is expected to be critically limiting when oxygen is scarce. Within mitochondria, UQ may arise from the following pathways (compiled from the metabolic atlas [32] https://metabolicatlas.org/explore/Human-GEM/gem-browser/metabolite/MAM03103m) summarized in Figure 1: UQ synthesis, catabolism of branched-chain amino acids, proline dehydrogenases reversal, electron transfer flavoprotein system reversal, dihydroorotate dehydrogenase (DHODH) reversal, α-tocopheryl hydroquinone (α-TQH_2_) metabolism, oxidation of UQH_2_ by Complex III (CIII) and Complex II (CII) reversal. Some of them are only theoretical considerations, or exert a minor contribution or are not applicable due to pertaining constraints. Specifically, the provision of UQ through *de novo* synthesis cannot be a source for residual Complex I activity in severe hypoxia because it is an oxygen-dependent pathway. Catabolism of the branched-chain amino acids — valine, leucine and isoleucine — takes place only in specialized tissues expressing the enzymes isovaleryl-CoA dehydrogenase and acyl-CoA dehydrogenase short/branched chain being also dependent on the input of 3-methylcrotonyl-CoA and tiglyl-CoA, respectively; the concentration of these thioesters is kept very low because of their tendency to inhibit important metabolic pathways such as synthesis of *N*-acetylglutamate, an activator of the first step in the urea cycle [33]. The reversibility of DHODH, proline dehydrogenases and the electron transfer flavoprotein system in oxidizing UQH_2_ are theoretical and/or can be demonstrated only under strictly artificial conditions, thus bear little — if any — pathophysiological *in vivo* relevance. Oxidation of UQH_2_ to UQ by α-TQH_2_ (possessing vitamin E activity) [34] may occur, but it only applies with this particular exogenous quinone [35]. Thus, the only viable possibilities for UQ provision during severe hypoxia are CIII activity — but only in the presence of a suitable electron acceptor — and CII reversal, addressed below.

## Complex III activity in hypoxia

**Figure 2.**
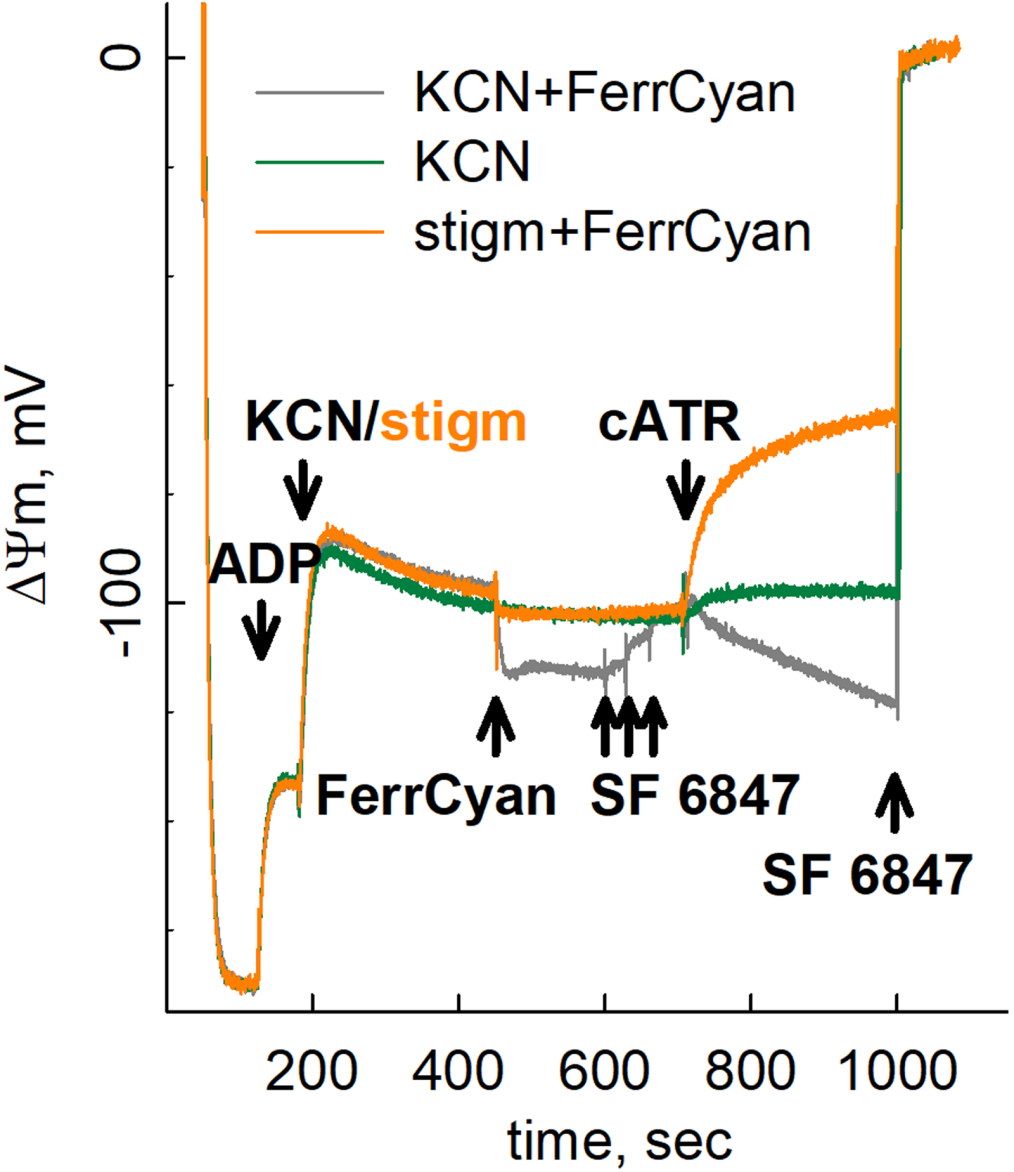
Ferricyanide sustains ANT forward mode operation in CIV- but not CIII-inhibited mitochondria. Reconstructed time courses of safranin O signal calibrated to ΔΨm in isolated mouse liver mitochondria, in the presence of glutamate and malate. Effect of ferricyanide (FerrCyan) on cATR-induced changes of ΔΨm after Complex III inhibition by stigmatellin (stigm) or Complex IV by KCN. Additions were as indicated by the arrows. At the end of each experiment, 1 μM SF 6847 was added to achieve complete depolarization. Reproduced by permission from [38].

CIII catalyzes the transfer of electrons from UQH_2_ to cytochrome c, coupled to the translocation of four H^+^ across the mitochondrial inner membrane. In turn, the electrons of the reduced cytochrome c are used to reduce oxygen by Complex IV (CIV), which also pumps two H^+^ out of the matrix. Overall, CIII activity depends on the availability of the final electron acceptor, oxygen. Thus, in hypoxia, CIII activity can only be maintained if an alternative electron acceptor with a redox potential higher than that of cytochrome c (the latter being ∼0.22 V) is available. Such a compound is ferricyanide, [Fe(CN)_6_]^3−^ exhibiting a redox potential of ∼0.4 V [36] (tunable over a 2.1 V range [37]). Indeed, by using ferricyanide, we showed that in isolated mitochondria with pharmacologically blocked CIV, CIII was able to maintain oxidation of UQH_2_ to UQ. In turn, UQ would be used by CI to oxidize NADH to NAD^+^, which would allow the α-ketoglutarate dehydrogenase complex to maintain mtSLP yielding high-energy phosphates. The experimental setting underlying the above considerations is shown in Figure 2 (reproduced by permission from [38]). Figure 2 depicts the time courses of safranine O fluorescence calibrated to membrane potential (ΔΨm, in mV) of isolated mouse liver mitochondria respiring on glutamate and malate. Where indicated, state 3 respiration was initiated by ADP, causing a depolarization as expected, followed by blocking CIII or CIV by stigmatellin or KCN, respectively, both clamping ΔΨm to ∼−100 mV. Subsequently, the ANT inhibitor carboxyatractyloside (cATR) was added. A cATR-induced depolarization indicates that prior to the addition of this inhibitor, the ANT was operating in reverse, while repolarization indicates that it was working in forward mode [39]. In respiration-inhibited mitochondria, ANT directionality is tethered to mtSLP: if mtSLP is operational and yields ATP, the ANT works in forward mode and *vice versa* [40,41]. The presence of ferricyanide (FerrCyan) converted the cATR-induced change in ΔΨm from a depo- to a repolarization when the respiratory chain was inhibited by KCN (blocking CIV) but not stigmatellin (blocking CIII). This can only mean that ferricyanide allowed UQH_2_ to be oxidized by CIII, fueling CI with UQ which in turn would provide the α-ketoglutarate dehydrogenase complex with NAD^+^ and permit glutamate catabolism through mtSLP, yielding ATP in the matrix. The stepwise additions of SF6847 (an uncoupler) after the addition of ferricyanide but before cATR served the purpose of returning ΔΨm to the ∼−100 mV clamp, for reasons explained in [41].

Unfortunately, experiments with ferricyanide can only be performed with isolated mitochondria (or permeabilized cells) because this compound (and practically all based on transition metals belonging to the category of inorganic compounds that can oxidize cytochrome c) is not membrane-permeable. However, there are compounds that are membrane-permeable and are able to oxidize cytochrome, such as dihydroethidium [42]. Copper proteins (cupredoxins) and other electron transfer proteins expressed in plants and bacteria may also be able to oxidize cytochrome c [43] and they could theoretically be made to express in mammalian cells by standard genetic engineering [44]. Alternatively, exogenous amphiphilic UQs (denoted as *exo* → UQ′ in Figure 1 that by default are membrane-permeable) could be administered to fuel mitochondrial diaphorases activity that oxidize NADH to NAD^+^ [38]. The ensuing UQH_2_ would be re-oxidized by CIII, provided of course that a suitable final electron acceptor is available [45].

From the above considerations it is concluded that in hypoxia, CIII could re-oxidize UQH_2_ to UQ for CI to maintain NAD^+^ provision for α-ketoglutarate dehydrogenase complex, only if a suitable final electron acceptor is available. When such an electron acceptor is not available, CII operating in reverse is the only means of UQ source for CI.

## Reverse Complex II activity in hypoxia

**Figure 3.**
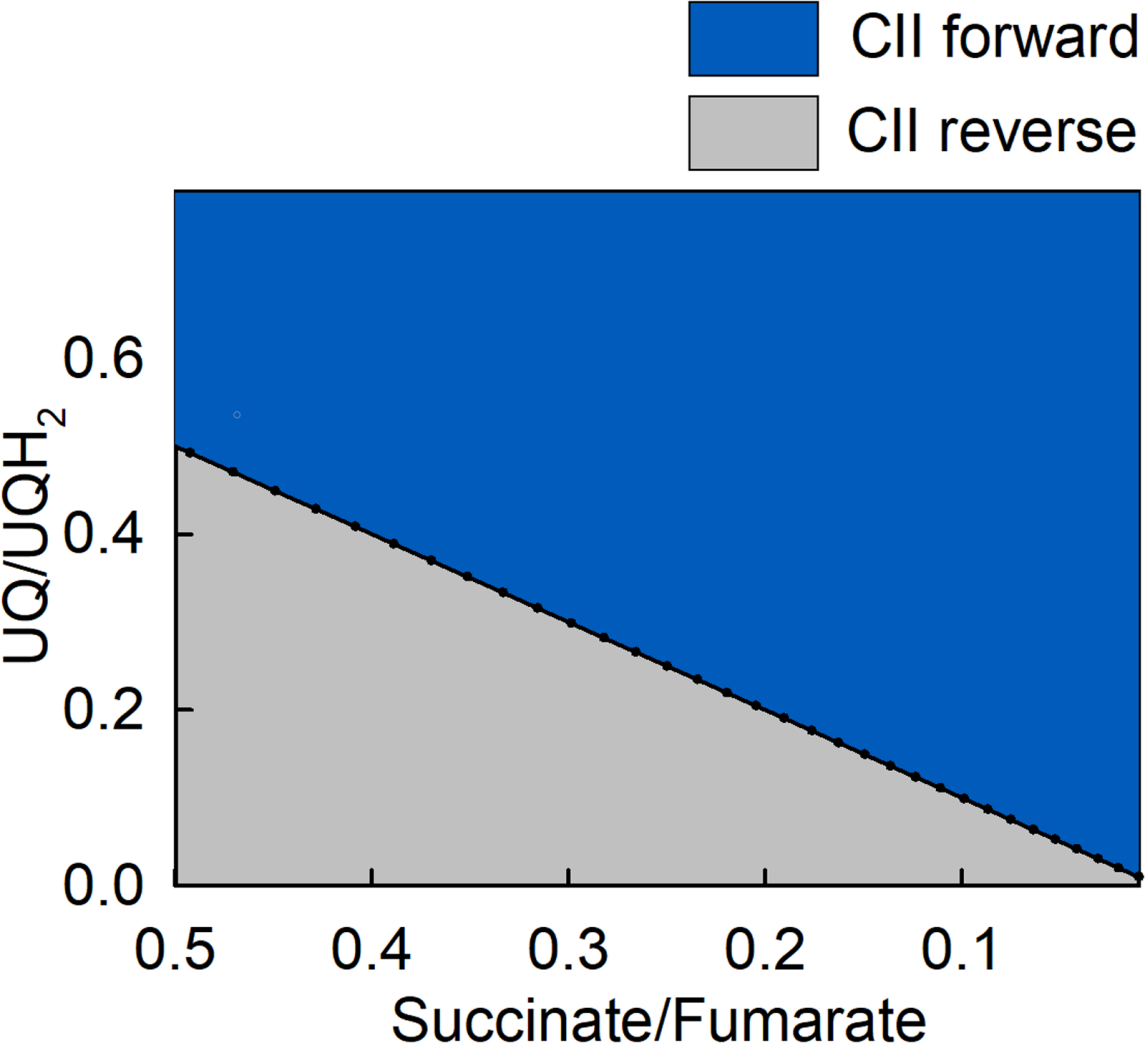
CII directionality. 2D plot of CII directionality depicted from succinate/fumarate ratio as a function of UQ/UQH_2_ ratio. Reproduced from [49].

In normoxia, CII catalyzes the oxidation of succinate to fumarate, reducing the covalently bound prosthetic group FAD to FADH_2_; in turn, FADH_2_ reduces UQ to UQH_2_, regenerating FAD [46]. The reversibility of the overall reaction has being extensively addressed for many reasons, reviewed in [47]. Current consensus is that CII operates in both forward and reverse mode in hypoxia [48], which we refer to as ‘amphidirectional’ [49]. When CII operates in reverse, fumarate is considered as the terminal electron acceptor [49,50], see also Figure 1. Despite that CII activity is subject to regulation [51,52], its directionality is governed by the succinate/fumarate and UQ/UQH_2_ pairs, see Figure 3 (reproduced from [49]). It is expected that changes in the concentration of reactants would sway CII directionality. To this end, it must be emphasized that [succinate] is influenced by succinyl-CoA ligase, [fumarate] by fumarase, [UQ] and [UQH_2_] by CI and other enzymes converging at the mitochondrial coenzyme Q junction [53]. Nevertheless, the take-home message is that in hypoxia, CII reverse activity provides UQ for CI. For that, the availability of fumarate is paramount. Fumarate enters mitochondria through SLC25A10 and SLC25A11 [54] at a very slow rate, thus if produced in the cytosol it is unlikely to contribute to CII reversal. On the other hand, within the mitochondrial matrix fumarate can be derived from malate — an abundant and highly transportable metabolite — through fumarase.

## NAD^+^ links Complex I to mtSLP during hypoxia

**Figure 4.**
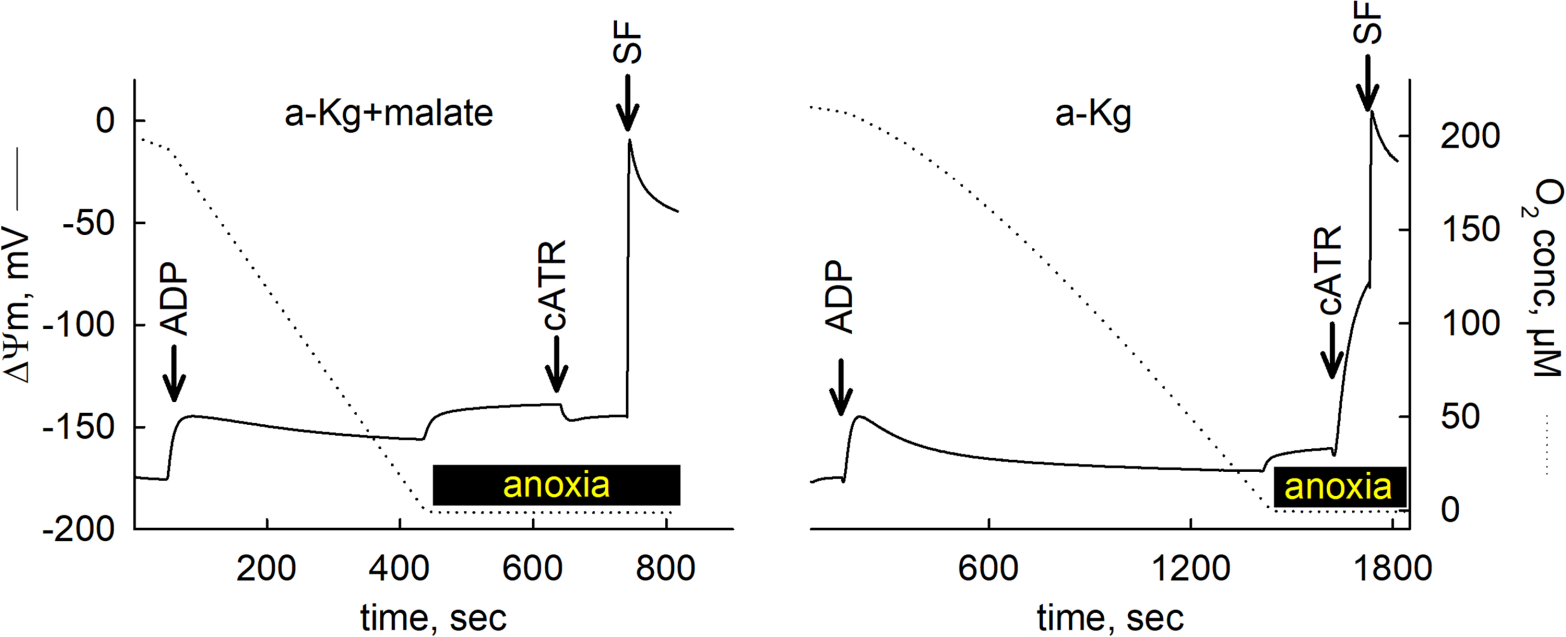
Malate supports ANT forward mode operation in anoxic mitochondria. Reconstructed time courses of safranin O signal calibrated to ΔΨm in anoxic isolated mouse liver mitochondria provided with either α-ketoglutarate and malate or only α-ketoglutarate. Additions were as indicated by the arrows. At the end of each experiment, 1 μM SF 6847 was added to achieve complete depolarization. Reproduced from [45].

**Figure 5.**
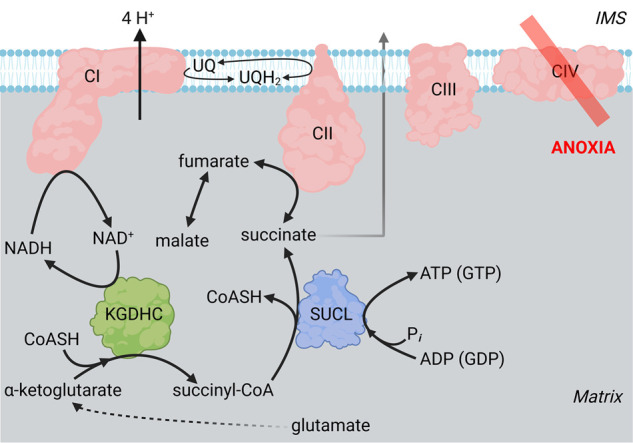
NADH oxidized by CI supports the oxidative decarboxylation of glutamate in anoxic mitochondria. Cartoon illustrating the oxidation of NADH by CI supporting α-ketoglutarate dehydrogenase complex (KGDHC), in turn maintaining the oxidative decarboxylation of glutamate during anoxia. The oxidative decarboxylation of glutamate (through α-ketoglutarate) leads to harnessing of the energy stored in the succinyl-CoA thioester to ATP (or GTP, depending on subunit composition) by succinyl-CoA ligase (SUCL). This process is referred to as ‘mitochondrial substrate-level phosphorylation’, mtSLP. CII operates in reverse or forward mode depending on the availability of fumarate (originating from malate). Succinate exits mitochondria so as not to sway the reversible SUCL reaction toward ATP (or GTP) hydrolysis.

From the above considerations it is irrefutable that during hypoxia, CII can operate in reverse providing UQ to CI, which is needed for oxidizing NADH. Having said that, it is important to emphasize that this scenario unfolds only if fumarate — most likely derived from malate — is available. Indeed, the co-presence of malate when α-ketoglutarate was present rescued mtSLP in anoxic mitochondria, see Figure 4 (reproduced from [45] by permission). This is because fumarate was derived, acting as final electron acceptor for a reverse-operating CII providing UQ to CI which oxidized NADH. Eventually, NAD^+^ would be used by the α-ketoglutarate dehydrogenase complex and maintain mtSLP, see Figure 5 (reproduced from [49]). mtSLP-derived ATP preserved ANT directionality in forward mode, hence the cATR-induced repolarization in the presence but not absence of malate. That α-ketoglutarate dehydrogenase complex is a critical node in this whole operation can be deduced from the effect of arsenite blocking this enzyme, where mtSLP in anoxic mitochondria supplemented by any NAD^+^-linked substrates plus malate, is abolished [49].

## mtSLP: harnessing oxidative decarboxylation of glutamine in cancer hypoxia

**Figure 6.**
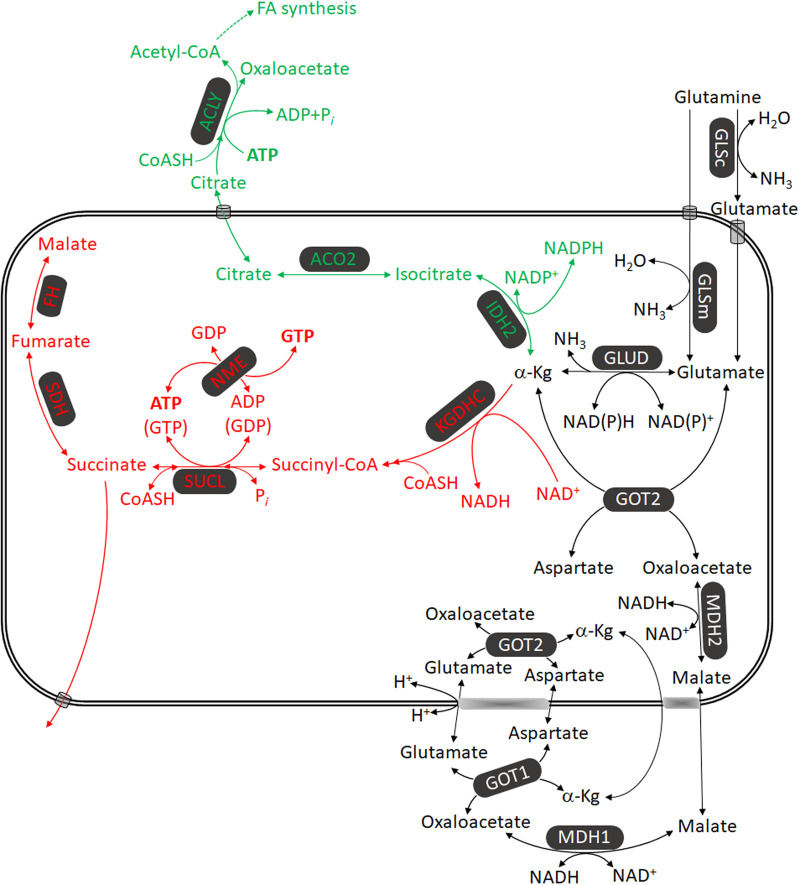
Oxidative decarboxylation and reductive carboxylation of glutamine. Glutamine metabolism through reductive carboxylation (green) and oxidative decarboxylation (red). α-Kg, alpha-ketoglutarate; ACO2, aconitase; ACLY, ATP citrate lyase; FA, fatty acid; GLSc, glutaminase, cytosolic; GLSm, glutaminase, mitochondrial; GLUD, glutamate dehydrogenase; GOT2, aspartate aminotransferase; IDH2, NADP^+^-dependent isocitrate dehydrogenase; KGDHC, alpha-ketoglutarate dehydrogenase complex; MDH1, malate dehydrogenase, cytosolic; MDH2, malate dehydrogenase, mitochondrial; NME, nucleoside diphosphate kinase; SDH, succinate dehydrogenase; SUCL, succinyl-CoA ligase. Reproduced from [58].

The ‘addiction’ of many tumors to glutamine (hence the inclusion of glutamine in most culture media known since the 1950s [55,56]) is sustained by up-regulating mechanisms involved in glutamine synthesis, autophagy-derived glutamine and uptake from extracellular sources [57]. In tumors, glutamine assumes roles in cell signaling, apoptosis, epithelial-to-mesenchymal transition, epigenetics and metabolism [57]. The metabolic fate of glutamine is oxidative decarboxylation (red pathway, Figure 6, reproduced from [58]) and catabolism through the citric acid cycle and/or reductive carboxylation (green pathway, Figure 6) toward fatty acid synthesis. According to the most recent human genome-scale metabolic model [59] glutamine participates in an additional >50 reactions that do not fall among these two branches, but are quantitatively far less important (omitted from Figure 6, for clarity). In the past few years it has become increasingly apparent that in many cancers, reductive carboxylation of glutamine toward fatty acid synthesis occurs with a greater flux than catabolism toward the citric acid cycle [60–63]. It is this author's opinion that lipid anabolism in cancer is a ‘venting’ mechanism to alleviate the associated reductive stress. Nevertheless, reductive carboxylation and oxidative decarboxylation are not mutually exclusive; in view of the very high rate of glutamine uptake to the cancer cell interior [64] it is expected that oxidative decarboxylation will also occur to an appreciable extent. The pathway glutamine → glutamate → α-ketoglutarate → succinyl-CoA →  succinate leads to the generation of high-energy phosphates through the reaction catalyzed by SUCL, i.e. mtSLP; regardless of the minor extent of ATP (or GTP, depending on SUCL subunit composition [65–67]) production compared with that by the mitochondrial F_0_–F_1_ ATP synthase, because it takes place within the mitochondrial matrix — a compartment with two-to-three orders of magnitude smaller volume than the cytosol — it will exert a two-to-three orders of magnitude greater effect in ATP (or GTP) concentration. This affects the directionality of ANT to the point that prevents it from importing ATP from the cytosol [39]. This is crucial, mindful of the adverse metabolic conditions frequently encountered in the tumor microenvironment encompassing low oxygen tension and scarce substrate availability leading to severe decreases in OXPHOS capacity [7,58] resulting in F_0_–F_1_ ATP synthase reversal which hydrolyzes ATP. The importance of mtSLP substantiated by SUCL rescuing cells during hypoxia has been shown in different settings, reviewed in [7].

## Constraints and concluding remarks

It has been shown that CI may exist in two forms: an active (*A*) and a de-active (*D*) form, with the latter signifying a dormant state of the complex, not inactivated or covalently modified [68]. The transition from the *A* to *D* occurs when substrate and oxygen availability are limited [69]. Currently, it is impossible to determine if the residual CI activity observed in [49] during hypoxia will be affected by the *A* to *D* transition. More recently, it was reported that the α-ketoglutarate dehydrogenase complex directly associates with CI [70]. It is unknown if this direct association plays a role, if any, in the mechanistic coupling of CI and the α-ketoglutarate dehydrogenase complex through NAD^+^ in hypoxia examined hereby. Based on the considerations reviewed above, it is concluded that CI serves as a drug target even when OXPHOS is compromised. This is due to its ability to supply NAD^+^ to the oxidative decarboxylation branch of glutamine catabolism, maintaining mtSLP and allowing the adenine nucleotide translocator (ANT) to operate in the forward mode, thereby un-straining cytosolic ATP reserves. Thus, despite the recent disappointing results of clinical trials using complex I inhibitors for treating cancer, there is potential for a therapeutic strategy. This may involve lower levels of CI inhibition in combination with targeting other sites within glutaminolysis, to disrupt cancer cell metabolism.

## Perspectives

• Addressing Complex I activity in hypoxia is pivotal for unraveling oncometabolic pathways, with NAD^+^ emerging as a key factor in sustaining cancer cell survival.

• Current state-of-the-art prompts for reevaluating the link between cancer cell survival and OXPHOS, emphasizing Complex I and mitochondrial substrate-level phosphorylation as focal points in understanding cancer metabolism.

• Future research should explore lower levels of Complex I inhibition and combination therapies targeting glutaminolysis nodes, providing avenues to overcome drug toxicities and refine cancer treatment strategies.

## Abbreviations

α-TQH_2_: α-tocopheryl hydroquinone
ANT: adenine nucleotide translocase
cATR: carboxyatractyloside
CI: Complex I
CII: Complex II
CIII: Complex III
CIV: Complex IV
DHODH: dihydroorotate dehydrogenase
mtSLP: mitochondrial substrate-level phosphorylation
OXPHOS: oxidative phosphorylation
UQ: ubiquinone
UQH2: ubiquinol
